# Sex Disparity in Systemic Sclerosis-Associated Pulmonary Fibrosis

**DOI:** 10.3390/ijms27104363

**Published:** 2026-05-14

**Authors:** Audrey N. Galimba, Ludivine Renaud, Samantha E. Kotz, Erica L. Herzog, Carol Feghali-Bostwick

**Affiliations:** 1 Division of Rheumatology and Immunology, Department of Medicine, Medical University of South Carolina, Charleston, SC 29425, USA; galimba@musc.edu (A.N.G.); renaudl@musc.edu (L.R.); sam29464@icloud.com (S.E.K.); 2 Yale-ILD Center of Excellence, Department of Medicine, Yale School of Medicine, New Haven, CT 06520, USA; erica.herzog@yale.edu

**Keywords:** systemic sclerosis, scleroderma, pulmonary fibrosis, sex disparity, RNA sequencing, transcriptomics, comparative analysis, fibroblasts

## Abstract

Systemic sclerosis (SSc) is a fibrotic disease with high mortality. SSc-associated pulmonary fibrosis (SSc-PF) is currently the leading cause of death. SSc shows a significant sex disparity, with an average sex ratio of 1:5 men to women, yet SSc-PF is more severe in men. We compared gene expression profiles of SSc-PF lung tissues from male and female donors. Whole lung tissues from healthy donors and SSc-PF patients of both sexes were analyzed by RNA sequencing. Selected genes were validated by quantitative polymerase chain reaction and Western blotting analyses. Our results show that genes related to extracellular matrix production were upregulated in females, while genes that are less explicitly related to fibrosis were upregulated in males. Additionally, recombinant transforming growth factor beta (TGFβ) elicited a different response in female and male normal lung fibroblasts. Further, the transcriptomic signatures in male and female lungs only overlapped by 9.19%, highlighting that SSc-PF progresses using different pathways in individuals of different sex. Furthermore, this sex-specific signature of SSc-PF highlights the importance of precision medicine when considering disease-modulating therapies.

## 1. Introduction

Systemic sclerosis (SSc) is an autoimmune connective tissue disease whose hallmark is fibrosis [1,2]. SSc has one of the highest mortality rates among rheumatic conditions, with the leading cause of death being SSc-associated interstitial lung disease [3]. Currently, Food & Drug Administration (FDA)-approved therapies for SSc-associated pulmonary fibrosis (SSc-PF) are limited and only slow the progression of PF. No current therapies can stop or reverse the disease, highlighting the need for more effective therapies [4,5,6].

Sex disparity is well-established in SSc, with a sex ratio ranging from 1:3 to 1:8 (male:female) [7,8,9]. However, men with SSc are significantly more likely to develop PF compared to women, with a prevalence up to 63% in men, compared to 49% in women [10]. Because of this, men with SSc have an increased overall mortality rate [7,8,9,11]. These differences may suggest a divergence in the pathogenesis of SSc between men and women.

Fibroblasts are considered the effector cells in fibrosis as their primary function is to maintain tissue homeostasis via production and maintenance of the extracellular matrix (ECM) [12,13]. They also play a supportive role in angiogenesis, inflammation, and wound healing [12]. Many growth factors can activate the profibrotic response of fibroblasts, including transforming growth factor β (TGFβ), connective tissue growth factor, and platelet-derived growth factor (PDGF). Chronic activation of fibroblasts via these and other factors contribute to the development of progressive fibrosis, as seen in SSc [6,12]. Profibrotic and antifibrotic processes in lung tissue are driven by a finely tuned balance of autocrine and paracrine signals secreted by a variety of cell types. For example, in the lung, TGFβ, a major fibrotic mediator, is predominantly produced by macrophages and epithelial cells [14,15]. Notably, while differences in gene expression by tissue have previously been cataloged [16], studies on the sex disparity in gene expression in the context of fibrotic SSc lungs and fibroblasts are critically lacking.

The goals of this study are to: (1) characterize the transcriptomic signature of SSc-PF whole lungs from male and female patients, identify similarities and differences by sex via comparative analysis, and define the differentially expressed genes (DEGs) and enriched pathways that are unique to males and females, (2) determine if differences exist in healthy lungs from male and female donors, (3) identify differences between SSc-PF lungs based on sex, (4) examine whether there is a differential response to TGFβ in normal lung fibroblasts from female and male donors, and (5) investigate whether potential therapeutics for SSc-PF may differ by sex. Ultimately, our study aims to identify and validate DEGs that are unique to female and male SSc-PF lungs to enhance our understanding of the pathogenesis of SSc in each sex and inform the development of tailored therapies.

## 2. Results

### 2.1. How Does the Gene Expression Profile of SSc-PF Compare in Females and Males?

**Figure 1:**
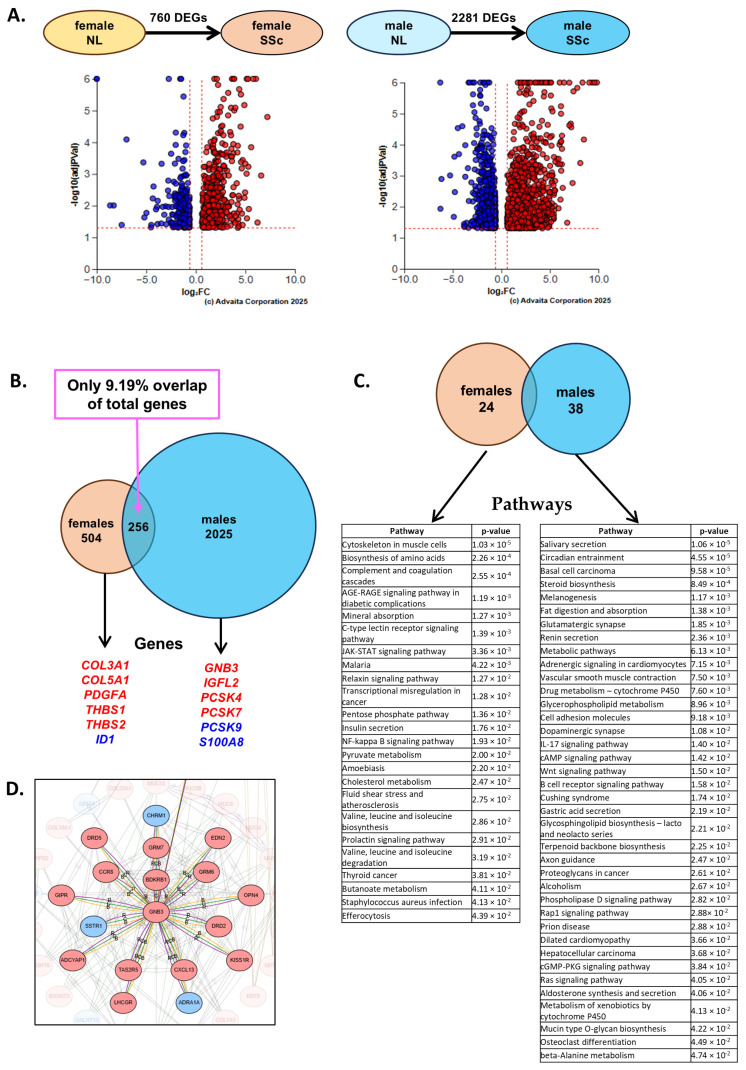
Comparison of SSc-PF progression in males and females (n = 6/group). (**A**) Volcano plots of the F-NL vs. F-SSc and M-NL vs. M-SSc comparisons. (**B**) Venn diagram of DEGs identified in the F-NL vs. F-SSc and M-NL vs. M-SSc comparisons outlining chosen genes of interest. Upregulated genes are shown in red, and downregulated genes are shown in blue. (**C**) Venn diagram and lists of pathways significantly perturbed by the DEGs in the F-NL vs. F-SSc and M-NL vs. M-SSc comparisons. (**D**) Hub gene diagram for the M-NL vs. M-SSc comparison generated by Advaita. GNB3 has a centrality degree of 1.

We first identified the DEGs in SSc compared to normal lung (NL) tissues of females (F) and males (M) by performing a differential expression analysis (DEA) “SSc vs. NL” in each sex (F-SSc vs. F-NL or M-SSc vs. M-NL) (q < 0.05, log2FC > |0.6|). The DEA returned 760 DEGs in females and 2281 DEGs in males (Figure 1A, Supplemental Files S1–S3). We then performed a comparative analysis, integrating the two RNAseq analyses to identify the DEGs and enriched pathways that are unique to females or males (Figure 1B,C, Supplemental Files S4 and S5). Five-hundred-and-four DEGs enriching 24 pathways were unique to females, and 2025 DEGs enriching 38 pathways were unique to males. Interestingly, only 9.19% of the DEGs overlapped between females and males (Figure 1B, Supplemental File S6).

**Figure 2:**
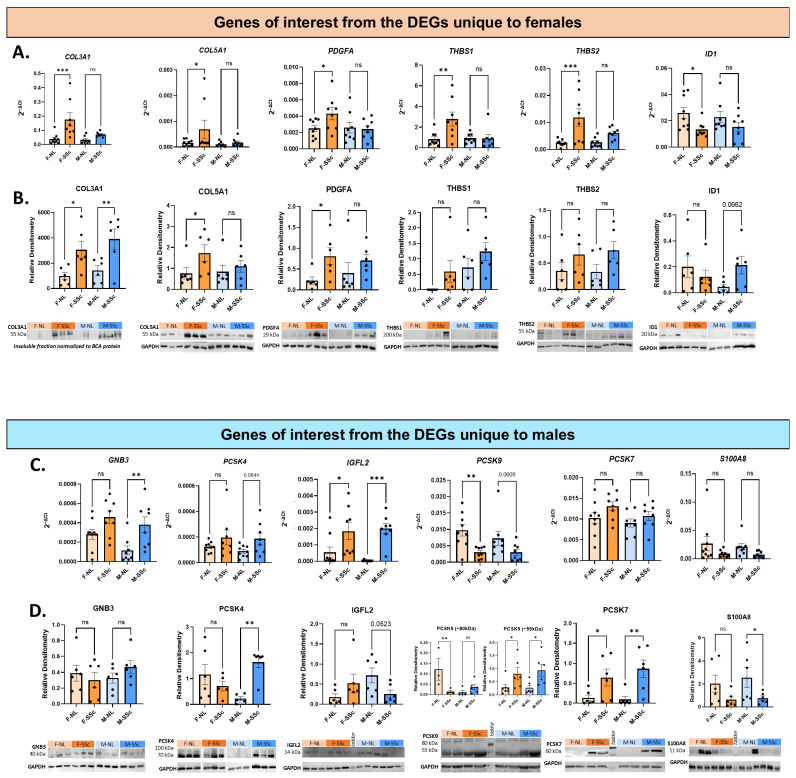
Validation of SSc-PF genes of interest in males and females. (**A**) RNA (n = 9/group) and (**B**) protein (n = 6/group) expression levels of GOIs from the DEGs unique to females: *COL3A1*, *COL5A1*, *PDGFA*, *THBS1*, *THBS2*, and *ID1*. (**C**) RNA (n = 9/group) and (**D**) protein (n = 6/group) expression levels of GOIs from the DEGs unique to males: *GNB3*, *PCSK4*, *IGFL2*, *PCSK9*, *PCSK7*, and *S100A8*. Representative Western blots are shown. For COL3A1, the insoluble fraction was analyzed by immunoblotting and normalized to BCA total protein. For all other selected targets, whole lung lysates were analyzed and normalized to GAPDH as a loading control. Gels and blots were processed in parallel. One-way ANOVA; Fischer–LSD test; ns, not significant; * *p* ≤ 0.05; ** *p* ≤ 0.01; *** *p* ≤ 0.001. Mean ± SEM. F = female; M = male.

In each list of unique DEGs, we selected several genes of interest (Figure 1B), including the hub gene “G protein subunit beta 3” (GNB3) that was significantly upregulated in males only and had the highest centrality degree score of “1” (Figure 1D, Supplemental File S7). The mRNA levels and protein abundance of all selected genes were measured by quantitative polymerase chain reaction (qPCR) and Western blotting, respectively, in whole lung homogenates (Figure 2).

#### 2.1.1. Differential Targets in Females (F-SSc vs. F-NL)

The mRNA data of all the female genes of interest selected for validation by qPCR were consistent with the RNAseq data, confirming that *COL3a1*, *COL5a1*, *PDGFA*, *THBS1* and *THBS2* mRNA levels were upregulated while *ID1* was downregulated in female SSc lungs (Figure 2A). We also validated our targets at the protein level (Figure 2B). The protein abundance was consistent with mRNA levels (Figure 2A) for COL3A1, COL5A1 and PDGFA, as all showed a significant increase in protein levels in female SSc lungs as compared to female NL. For COL3A1, the data also captured a significant increase in male SSc lungs compared to male NL, a trend that was observed at the mRNA level but did not reach significance (Figure 2B). Due to the variability in the dataset, significance was not reached for THBS1, THBS2 and ID1, but a clear trend mirroring the mRNA levels was observed, showing an increase in protein abundance for THBS1 and THBS2, and a decrease in ID1 in the female SSc lungs. In male SSc lungs, a noticeable increase in these three targets was observed, especially for ID1 (*p* = 0.0662), an unexpected result since this increase was not observed at the mRNA levels in male lung tissues (Figure 2A). In addition a significant sex × disease interaction was detected for IGFL2 protein levels (*p* = 0.0191).

#### 2.1.2. Differential Targets in Males (M-SSc vs. M-NL)

For DEGs of interest selected from the “unique to males” list, the qPCR data revealed a layer of regulation that was not always captured by the RNAseq data (Figure 2C). For *GNB3* and *PCSK4*, the qPCR data was consistent with RNAseq data as both were only upregulated in male SSc lungs, although the significance threshold was not reached for *PCSK4* (*p* = 0.0644) despite a noticeable increase in male SSc lungs compared to NL. For *IGFL2* and *PCSK9*, a significant deregulation was observed in both male and female SSc lungs (upregulation for *IGFL2* and downregulation for *PCSK9*), whereas the RNAseq data showed that deregulation occurred only in males. Lastly, the data for *PCSK7* and *S100A8* showed trends but no significant changes by qPCR despite being DEGs in the RNAseq dataset. At the protein level (Figure 2D), PCSK4, PCSK7, and S100A8 protein abundance matched the mRNA trends in males, albeit not significantly for PCSK4 (*p* = 0.0735). However, the protein data for GNB3, IGFL2, and PCSK9 were inconsistent with their mRNA levels. Overall, there is very little overlap in the molecular signature of female and male SSc-PF tissues, marked by the sex-specific perturbation of pathways such as “cytoskeleton in muscle cells,” “biosynthesis of amino acids,” and “complement and coagulation cascades” in females and “salivary secretion,” “circadian entrainment,” and “basal cell carcinoma” in males (Supplemental File S3).

#### 2.1.3. Fibroblast Markers in SSc-PF in Females and Males

On a cellular level, Tsukui et al. [17] showed that, in response to an injury, alveolar fibroblasts differentiate into distinct subpopulations: inflammatory or fibrotic fibroblasts, and each group has distinct molecular markers. Strikingly, in the male SSc-associated transcriptomic changes, seven alveolar fibroblast markers are significantly downregulated (*INMT*, *AOC3*, *FMO2*, *TCF21*, *GPM6B*, *SCN7A*, and *GPX3*), while one fibrotic (*TNC*) and six inflammatory fibroblast markers (*CXCL14*, *SFRP2*, *SFRP4*, *PTGDS*, *LMNA*, and *TGFBI*) are significantly upregulated (Supplemental File S2). Meanwhile, our cross-sectional disease comparison in females yielded five upregulated inflammatory (*SERPINE1*, *SFRP4*, *BGN*, *DPT*, and *LMNA*) and four upregulated fibrotic fibroblast markers (*CTHRC1*, *COL1A1*, *COL1A2*, and *COL3A1*) (Supplemental File S1).

### 2.2. Comparison of M-SSc and F-SSc Lung Tissues

Differences in disease state were also examined by running the DEA “males SSc vs. females SSc”. This analysis returned only 35 DEGs in male compared to female SSc lungs (Supplemental File S11). Each of the perturbed pathways—”PPAR signaling”, “ferroptosis”, “coronavirus”, “transcriptional misregulation in cancer”, “riboflavin metabolism”, “rheumatoid arthritis”, “phagosome”, and “cholesterol metabolism”—only has one or two DEGs contributing to their perturbation (Supplemental File S12), further demonstrating that female and male SSc-PF lungs share a similar transcriptome, despite the differences captured in the comparative analysis. These results are unexpected since there was only a 9.19% overlap in lung tissue DEGs (Figure 1A), and we were anticipating to find significant differences in SSc-PF lungs of females and males. Instead, these results suggest that even though the disease process may be different in females and males, they converge to a similar transcriptomic phenotype in SSc-PF lungs at transplant, regardless of sex.

### 2.3. Comparison of M-NL and F-NL Lung Tissues

**Figure 3:**
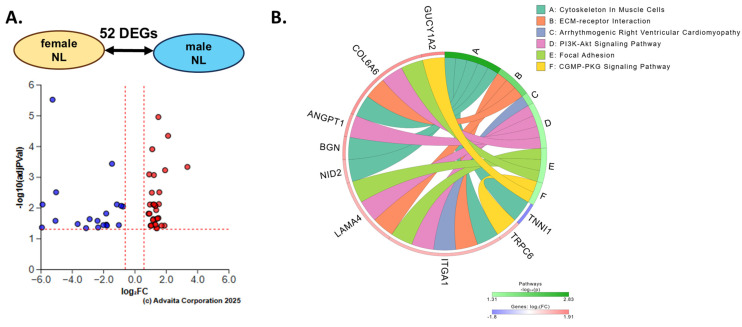
Comparison of F-NL and M-NL (n = 6/group). (**A**) Volcano plot of DEGs in the F-NL vs. M-NL comparison. Upregulated genes are shown in red, and downregulated genes are shown in blue. (**B**) Diagram of the top nine DEGs influencing the top six predicted pathways in the F-NL vs. M-NL comparison.

To identify differences in non-fibrotic lungs of male and female donors, the DEA “male NL vs. female NL” was performed and returned 52 DEGs (Figure 3A), along with the enrichment of six Kyoto Encyclopedia of Genes and Genomes (KEGG) pathways (Figure 3B, Supplemental File S8). Detectable but non-significant increases in COL6A6 and ANGPT1 (Supplemental File S9) proteins were seen in male NLs compared to female NLs. The upregulation of *COL6A6*, *ANGPT1*, *LAMA4* and *ITGA1* in male NLs as compared to female NLs contributed to the enrichment of “ECM–receptor interaction”, “PI3K-Akt signaling pathway” and “focal adhesion” pathways (Supplemental Files S9 and S10). Using Advaita’s proprietary Impact Analysis, KEGG figures were generated, showing the combined effect of the upregulation of these four genes and the predicted downstream effects on these three enriched pathways (Supplemental Files S9 and S10).

Looking at the “ECM–receptor interaction” pathway (Supplemental File S9B), the upregulation of *COL6A6*, *LAMA4* and *ITGA1* is predicted to upregulate several integrin subunits, including *ITGA2* (α2), *ITGA3* (α3), *ITGA6* (α6), *ITGA7* (α7), *ITGAV* (αV), *ITGA9* (α9), *ITGA10* (α10), *ITGA11* (α11), *ITGB1* (β1) and *ITGB4* (β4), as well the glycoprotein VI platelet (*GPVI*), two syndecans (*SDC1*, *SDC4*), *CD44*, three synaptic vesicle glycoproteins 2 (*SV2A*, *SV2B*, *SV2C*), and dystroglycan 1 (*DAG1* listed under αDG/βDG).

In the “PI3K-Akt signaling pathway” (Supplemental File S10A), the upregulation of *ANGPT1* is predicted to activate several receptor tyrosine kinases (RTKs), leading to cell proliferation, angiogenesis, and DNA repair via the RTK/MEK/ERK axis. It is also predicted to upregulate levels of *IRS1* and members of the Class IA PI3Ks. This is reinforced by the upregulation of *COL6A6*, *LAMA4* and *ITGA1* because this signature is also projected to upregulate the PI3Ks.

The combined upregulation of *COL6A6*, *LAMA4* and *ITGA1* also has an impact on the “focal adhesion” pathway (Supplemental File S10) as it would upregulate ITGBs, causing the activation of *SRC*, the focal adhesion kinase (FAK) *PTK2*, and the axis SHCs/RAF1, ultimately leading to the downregulation of the *BCL2* associated agonist of cell death (BAD). Additionally, our data suggest that male normal lungs may be in a “pre-fibrotic” state, primed for a response to a stimulus such as TGFβ. Thus, we next examined the response of female and male NL fibroblasts to TGFβ1.

### 2.4. Response of Female and Male Lung Fibroblasts to TGFβ Stimulation

**Figure 4:**
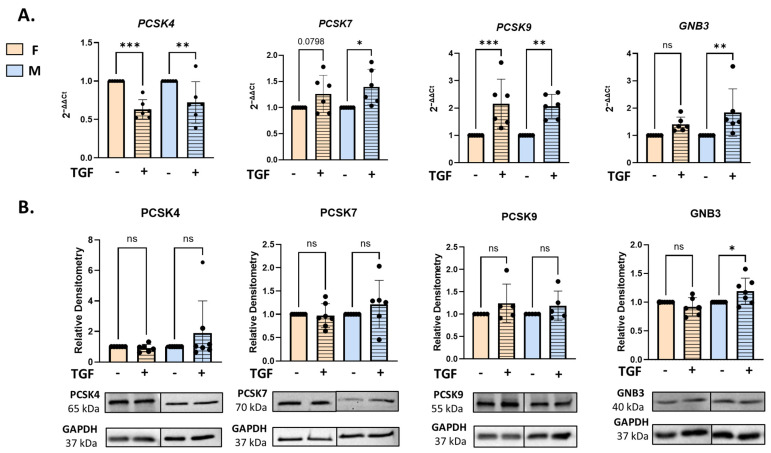
Differential expression analysis between F-NL and M-NL fibroblasts treated with TGFβ over 24 h (n = 6/group). (**A**) RNA expression levels of *PCSK4*, *PCSK7*, *PCSK9*, and *GNB3* in F- and M-NL fibroblasts treated with TGFβ for 24 h. (**B**) Protein abundance of PCSK4, PCSK7, PCSK9, and GNB3 in TGFβ-treated F-NL fibroblasts compared to M-NL fibroblasts, normalized to GAPDH loading control and representative Western blots for each protein. Samples derive from parallel experiments, and gels and blots were processed in parallel. One-way ANOVA, Fischer-LSD test, ns, non-significant; * *p* ≤ 0.05, ** *p* ≤ 0.01, *** *p* ≤ 0.001. Mean ± SEM.

To examine whether female and male fibroblasts differ in their initial response to a defined profibrotic stimulus, we recorded the response of selected genes to 24 h of TGFβ1 stimulation in NL fibroblasts. *PCSK4* mRNA levels decreased in NL fibroblasts of both sexes. Expression levels of *PCSK7* and *PCSK9* increased in both sexes, while *GNB3* levels increased significantly in male NL fibroblasts and showed an increasing trend in female NL fibroblasts. (Figure 4A). After 24 h of TGFβ1 stimulation, only GNB3 protein levels showed a significant increase at the protein level in male fibroblasts only (Figure 4B). PDGFA and THBS1 increased as expected in both sexes (Supplemental File S13). Overall, our data show that even at early timepoints after TGFβ stimulation, there is a differential response by sex in the expression of some of our target genes, particularly *THBS2* in females and *GNB3* in males.

### 2.5. Leveraging Sex-Based Differential Gene Expression Profiles for the Identification of Potential Therapies

**Figure 5:**
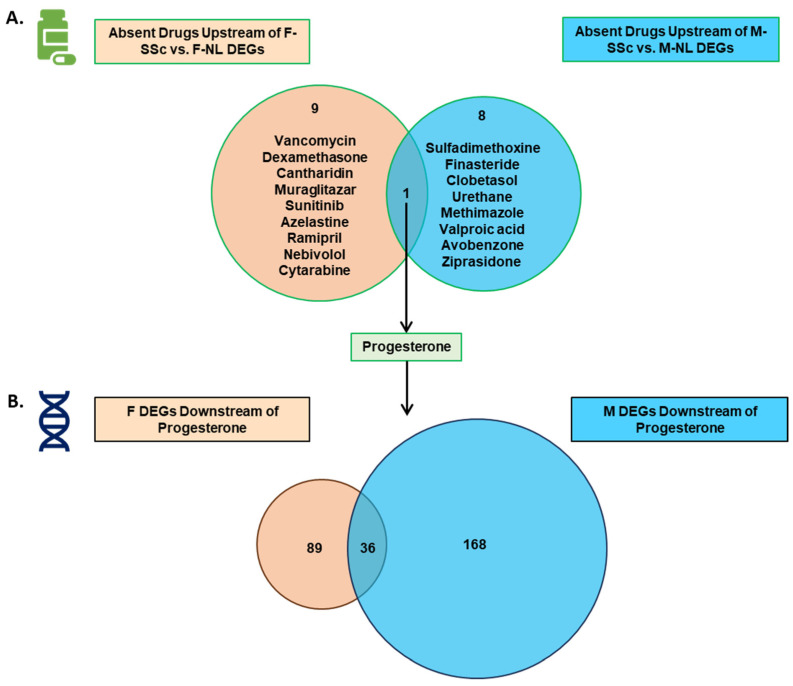
Drugs predicted to be absent upstream of SSc DEGs vary by sex. (**A**) Venn diagram comparing the chemicals predicted to be absent from the comparison “F-SSc vs. F-NL” to the DEGs obtained from the comparison “M-SSc vs. M-NL” (adjusted *p*-value < 0.05). At the intersect, only progesterone is commonly predicted to be absent in F-SSc and M-SSc lungs. (**B**) DEGs downstream of progesterone from the F-SSc vs. F-NL and M-SSc vs. M-NL comparisons.

Our data show that the gene expression profile in male and female SSc-PF lung tissues is quite different, with only a 9.19% overlap. Therefore, the transcriptomic signature of both sexes should be considered when developing potential therapies. To explore this, we used the “Upstream Regulators” tool provided by iPathwayGuide that assumes that if a drug’s effects are negatively correlated with SSc-associated DEGs, that compound would be a good candidate to revert the differential expression profile in SSc and potentially suppress the phenotype. This in silico analysis identifies these “absent drugs” for potential repurposing as SSc-modulating drugs. By comparing the drugs that are “predicted to be absent” between the groups (Figure 5A), 10 drugs upstream of the female DEGs and 9 upstream of the male DEGs were identified (Figure 5A). Only progesterone was predicted to impact the SSc-PF molecular signature in both sexes. However, of the DEGs downstream of progesterone, only 12.3% (36 DEGs) are common in the female and male analysis, leaving 89 and 168 downstream female-only and male-only DEGs, respectively (Figure 5B, Supplemental File S14).

By analyzing which genes have the greatest centrality via an adjacency matrix (Supplemental Files S15 and S16) [18], we can identify the unique signature of female and male SSc-PF genes that are targeted by drugs that are already FDA-approved. Notably, in females, the most centrally affected genes included *PDGFA*, *NDRG2*, and *VCAM1* (Supplemental File S15). In males, the central fingerprint included *FOXA2*, *CUX2*, and *MEOX1* (Supplemental File S16). The adjacency matrices were used to estimate which drugs would have the largest impact on the DEGs (Supplemental File S17). The top three drugs were progesterone (affecting 24.8% of F DEGs), sunitinib (20.0%), and dexamethasone (19.6%) in the female comparison, and valproic acid (affecting 36.4% of M DEGs), progesterone (10.1%), and urethane (9.4%) in the male comparison.

**Figure 6:**
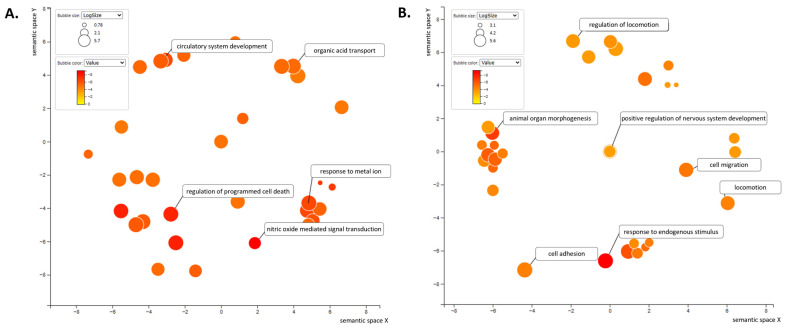
Defining unique biological processes targetable by drug repositioning. GO terms obtained from ToppFun for the 24 DEGs impacted by three or more female-specific drugs (**A**) and 89 DEGs impacted by three or more male-specific drugs (**B**) were entered in REViGO. The scatter bubble plots for Biological Process terms are shown here. Selected enriched terms are labeled in the scatterplot. The bubble color indicates the log_10_ *p*-value obtained from the ToppFun output. Bubble size indicates the frequency of the GO term in the underlying database, and thus more general terms are larger. The coordinate axes have no intrinsic meaning as REViGO uses multi-dimensional scaling to reduce the dimensionality of a matrix of the GO terms’ pairwise semantic similarities.

Despite nearly a four-fold greater number of DEGs being observed downstream of these potential drugs in males compared to females, Gene Ontology (GO) analysis revealed only two Molecular Function terms (TNF receptor binding and TNF receptor superfamily binding) and no Cellular Component terms associated with the core downstream gene signature in males (Supplemental File S18). In contrast, the female signature revealed 35 Cellular Component and Molecular Function terms each (Supplemental File S19). Analysis of the Biological Process category impacted by the female and male drugs revealed apoptosis, response to metals, and circulatory system development as the most impacted overarching themes in females (Figure 6A), compared to responses to endogenous stimuli, embryonic development, and cell locomotion in males (Figure 6B). Overall, we found that there is only one drug—progesterone—that is predicted to modulate the cross-sectional disease signature of SSc-PF in males and females, and that there is a unique signature of genes that may provide selective targets when considering sex-specific therapeutics.

## 3. Discussion

We identified a divergence in the molecular signature of disease by sex which has not been previously reported in SSc-PF lung tissues. Our findings suggest that the potential trajectory to severe SSc-PF varies significantly by sex, but that in late-stage disease, female and male SSc-PF lungs are transcriptomically similar. At the same time, control donor male lungs at baseline show enrichment of pro-fibrotic pathways when compared to female lungs, and normal lung fibroblasts respond differently to TGFβ depending on the sex of the donor. Finally, drugs that are predicted to impact the trajectory of disease vary significantly by sex, and the signature of genes affected by these repositionable drugs suggests that the most effective therapeutic targets and therapies themselves may be sex-specific.

### 3.1. There Is a Less than 10% Overlap in SSc-PF Disease Signature in Females and Males

Male SSc patients have a later disease onset, a greater likelihood of diffuse SSc and severe pulmonary fibrosis, and greater morbidity and mortality than female patients [7]. There is very little overlap (9.19%) in the molecular trajectory between female and male SSc-PF lungs. In this analysis, female-specific DEGs, such as *COL3A1*, *COL5A1*, *THBS1*, and *THBS2*, are explicitly associated with fibrosis, while male-specific genes relate more broadly to cell signaling (*GNB3*), metabolism (*IGFL2*), protein processing (*PCSK4*, *PCSK7*, and *PCSK9*), and innate immunity (*S100A8*). This is particularly interesting given that, when analyzing cause of death in patients with SSc between sexes, SSc-PF was the leading cause of death in males but not females [7].

A shared deregulated pathway is PI3K-Akt signaling, consistent with prior reports. This pathway influences ECM regulation via fibroblast apoptosis, senescence, autophagy, glycolysis, and oxidative species modulation [19], and promotes myofibroblast differentiation independent of TGFβ signaling in SSc [20]. However, its enrichment in the current study appears to be driven by different genes in female compared to male SSc lungs.

#### 3.1.1. The Unique Transcriptome of F-SSc vs. F-NL

In female SSc lungs, PI3K-Akt signaling perturbation is driven by genes such as *PDGFA*—validated as upregulated in female disease—and *THBS1* and *THBS2*, which contribute to focal adhesion signaling via integrins. *PDGFA* encodes PDGF-A, forming PDGF-AA or PDGF-AB. PDGF-A is increased in the bronchoalveolar lavage fluid of SSc patients [21], supporting its role in the pathogenesis of the disease. Our current analysis shows that both *THBS1* and *THBS2* mRNA levels are elevated in female SSc lungs, with protein levels trending upward in both sexes. The thrombospondin (THBS) family includes five secreted matricellular glycoproteins [22,23]. THBS1, largely platelet-derived, participates in acute inflammatory repair, while THBS2, produced by fibroblasts and smooth muscle cells, contributes to later remodeling phases [24,25]. As downstream targets of dexamethasone, their altered expression suggests that glucocorticoids may modulate SSc-PF [26,27].

Female-specific genes converge on overlapping pathways, including “protein digestion and absorption”, “ECM–receptor interaction”, “focal adhesion”, and the previously discussed “PI3K-Akt pathway”. A uniquely perturbed pathway in female SSc is “cytoskeleton in muscle cells,” driven by *THBS1*, *THBS2*, and *COL5A1* upregulation via ECM–receptor interactions. Together, these genes form a coherent module involving ECM remodeling, TGFβ activation, and vascular proliferation, consistent with the dual pathology associated with SSc, involving both fibrosis and vasculopathy.

#### 3.1.2. The Unique Transcriptome of M-SSc vs. M-NL

In male SSc vs. NL lungs, *GNB3* emerged as a key DEG in SSc-PF. *GNB3* encodes the β3 subunit of heterotrimeric G-proteins that relay signals from the cell surface to intracellular pathways. While not typically linked to fibrosis, a *GNB3* C825T polymorphism has been associated with hypertension, obesity, pre-eclampsia, and depression [28,29,30]. Here, *GNB3* is upregulated in male SSc-PF, with increased protein abundance even 24 h after TGFβ stimulation, suggesting it may act as an early profibrotic response gene in males.

Our prior work showed *GNB3* enrichment in African American vs. European American SSc lung fibroblasts, contributing to the “GABAergic synapse pathway” [31]. In the current analysis, *GNB3* is also a downstream target of valproic acid, a GABAergic enhancer, which additionally regulates *IGFL2*, *PCSK7*, and *PCSK9*. While the role of GABAergic signaling in fibrosis is unclear, GABA (gamma-aminobutyric acid) reduces inflammatory cytokines such as IL6 and IL12 in antigen-presenting cells and macrophages [32]. IL6 is upregulated in SSc fibroblasts, and immune cell–fibroblast crosstalk is central to SSc pathogenesis [33,34]. As a hub gene, GNB3 contributes several top indicated pathways in males, including “circadian entrainment”.

“Circadian entrainment” was the second most perturbed pathway in the male NL vs. SSc-PF comparison. Chang et al. [35] found that collagen homeostasis depends on circadian regulation, and clock disruption led to abnormal collagen fibrils [35]. Rhythmic expression of *Npas2*, *Per1/2/3*, *Bmal1 (Arnt1)*, and *Cry1*, but not of fibrillar collagen genes, suggests circadian regulation of collagen translation. Our data show significant upregulation of *PER1* and *PER2* in male SSc-PF, potentially explaining the advanced fibrosis observed despite limited increases in collagen transcripts. Disrupted circadian and translational regulation may also underlie the inverse RNA–protein relationships seen for *ID1*, *IGFL2*, and *PCSK9.*

The proprotein convertase subtilisin/kexin (PCSK) family mediates key post-translational processing. PCSK4, PCSK7, and PCSK9 showed the strongest sex-specific differences in our analysis. PCSK4 activates secretory precursors and is essential in reproduction. Its link to fibrosis is emerging, with an imbalanced relationship between PCSK4 and insulin-like growth factor II (IGF2) in a study of solitary fibrous tumors [36]. IGF2 is known to be overexpressed in SSc-PF and promotes fibrosis by increasing collagen I, fibronectin, and TGFβ [37,38]. As a pro-IGF2 convertase [36], PCSK4 upregulation in male SSc-PF suggests a role in disease pathogenesis.

PCSK7 participates in metabolic and cardiovascular disease and processes pro-TGFβ1 in zebrafish, correlating with TGFβ1 expression [39]. Our study newly implicates PCSK7 in human fibrosis. PCSK9, known for cholesterol metabolism, influences fibrosis via Wnt/β-catenin signaling, and its inhibition reduces fibrosis in bleomycin models [40]. Here, PCSK4 and PCSK7 were upregulated in male SSc-PF, while *PCSK9* was downregulated; at the protein level, pro-PCSK9 decreased in female SSc lungs, but active PCSK9 increased in SSc lungs of both sexes. These findings indicate sex-specific regulation of the PCSK family and support their involvement in SSc-PF pathogenesis.

Some discrepancies between transcriptomic discovery and validation are expected due to assay sensitivity, the use of different methodologies, or post-transcriptional regulation. Genes with concordant RNA-seq, qPCR, and Western blot—*COL3A1*, *COL5A1*, *PDGFA*, and *PCSK4*—provide the strongest evidence for sex-specific mechanisms. Several others showed co-directional trends in validation despite not reaching RNA-seq significance, supporting the underlying biological signal. 

### 3.2. All Pathways Converge to the Same SSc-PF Transcriptomic Signature

Since the cross-sectional disease signatures differed markedly between females and males, we anticipated substantial sex-specific differences in SSc-PF. Instead, our results show that females and males ultimately converge on a similar transcriptomic phenotype in end-stage SSc-PF, with only 35 DEGs contributing to eight pathways distinguishing male and female SSc-PF lungs.

This pattern of early transcriptomic divergence followed by late convergence in SSc-PF has not been previously described. Clinical observations from the Scleroderma Lung Studies (SLS) I and II clinical trials suggested that sex differences in SSc outcomes may reflect distinct inflammatory and fibrotic mediators [8], which aligns with our findings. Considering the progression from alveolar to inflammatory to fibrotic fibroblasts, the sex-specific patterns of fibroblasts in SSc are notable. In males, downregulation of alveolar fibroblasts may indicate ongoing differentiation into inflammatory fibroblasts. In contrast, female SSc lungs show no perturbation of alveolar markers. This divergence may contribute to the greater clinical severity observed in male patients with SSc.

### 3.3. Pro-Fibrotic Pathways Are Enriched in Control Male Lungs

The enrichment of the “ECM–receptor interaction”, “PI3K-Akt signaling”, and “focal adhesion” pathways in the “M-NL vs. F-NL” comparison suggests that male normal lungs may be in a “pre-fibrotic” state, primed to respond to stimuli such as TGFβ. Among the 59 DEGs distinguishing male and female normal lungs, several have established roles in fibrogenesis, while others remain uncharacterized. The most significantly upregulated genes in males include *GUCY1A2*, *COL6A6*, *ANGPT1*, and *BGN*. *BGN*, encoding biglycan, is X-linked, which may have implications for sex-specific presentations. The molecule is involved in collagen fibril assembly, inflammation, and innate immunity, and has been proposed as a candidate biomarker for SSc in addition to being a marker of inflammatory fibroblasts [17,41]. Except for *ANGPT1*, our study is the first to report significant sex differences in the expression of these genes. The strong enrichment of “ECM–receptor interaction”, “PI3K-Akt signaling”, and “focal adhesion” pathways, all of which are implicated in fibrosis and SSc [19,42,43], may help explain sex-based clinical differences in SSc-PF and potentially other fibrotic diseases such as IPF. These transcriptomic differences highlight the variation that exists between healthy female and male lungs.

#### There Is a Sex-Specific Response to TGFβ in Normal Lung Fibroblasts

Our data show that even at early timepoints after TGFβ stimulation, there is a sex-specific transcriptional response, particularly for *THBS2* in females and *GNB3* in males. For genes that have a known relationship with TGFβ, such as *THBS1* and *PDGFA*, the degree of significance also varied by sex. The timing of stimulation is important, as some targets act early in disease progression and may only show deregulation at specific intervals [44]. Ultimately, our findings support that even at short exposure times (i.e., 24 h), male and female fibroblasts respond differently to a fibrotic stimulus, which may contribute to sex-specific clinical presentations in SSc-PF. This also emphasizes the importance of reporting fibroblast and other data by sex.

### 3.4. Only One Drug Is Predicted to Impact Disease Trajectory in Both Females and Males

We found that only progesterone is predicted to modulate the trajectory of SSc-PF in both males and females, with only 12.3% of progesterone-regulated DEGs being significant in both sexes. Beyond sex-shared effects, each sex displays a distinct gene signature that may offer focused targets for sex-specific therapeutics. The idea that there are different sensitivities and responses to therapies in patients between men and women with rheumatic disease is not new [45]. In SLS I and II, women but not men with SSc showed functional improvement when treated with cyclophosphamide or mycophenolate mofetil [8]. Many of the impactful drugs identified in our analysis are FDA-approved for other indications, suggesting potential for drug repositioning. Note that urethane, although historically used as an anesthetic or for hematologic conditions, is not FDA-approved and is considered a likely carcinogen.

The presence of dexamethasone in the female comparison and methimazole in the male comparison hint at compounds that may modulate the fibroblast phenotype, though steroids remain undesirable in SSc due to the risk of precipitating renal crisis. Dexamethasone is predicted to decrease the expression of pro-inflammatory and pro-fibrotic markers *COL1A1*, *BGN*, *COL3A1*, and *COL1A1*, while methimazole is predicted to increase the expression of alveolar markers *SCN7A* and *INMT* and decrease the expression of inflammatory markers *TGFBI* and *CXCL14*. A precision medicine approach to SSc-PF may therefore be critical in developing effective treatments for each sex, particularly in patients with a refractory disease on conventional therapy.

The dominant gene signatures significantly affected by the potential drugs differ sharply between sexes. GO analysis reveals that female-targeted genes are enriched for the biological processesses “regulation of apoptotic process”, “response to metal ion”, and “blood vessel development”. Meanwhile, male-targeted genes involve “response to endogenous stimuli”, “embryonic pattern specification”, and “cell migration”. These findings support that as a function of sex-specific mechanisms driving SSc-PF, precise therapies must be considered as they ultimately target significantly different molecular fingerprints. Importantly, the difference in the pathogenesis of SSc-PF in women and men suggests that effective, disease-modifying drugs may be sex-specific.

## 4. Materials and Methods

### 4.1. Lung Tissues

Lung tissues were obtained from donor controls and patients with SSc as previously described [38]. Control and SSc patient lung tissues included female and male donors, with ages ranging from 20 to 67 years old (yo) for healthy donors (NL) and from 26 to 68 yo for SSc-PF patients (SSc) (Table 1 and Table 2). For all experiments, an equal number of male and female donor samples were used. All SSc patients had pulmonary fibrosis (FVC ≤ 70%). In addition, one SSc patient had mild pulmonary hypertension (PA mean > 25 mmHg) and one had an unknown hypertensive status (SSc-19 and SSc-81, respectively).

### 4.2. Whole Lung Processing for RNA Extraction and Immunoblotting

Five-millimeter punches of whole lung sections were flash-frozen and stored at −80 °C. Tissue was homogenized in the bead ruptor (Omni International, Kennesaw, GA, USA) at 6950 rpm for 30 s four times in either Trizol (Qiagen, Germantown, MD, USA) for RNA extraction, or in RIPA buffer with 1× halt protease inhibitor (ThermoFisher Scientific, Waltham, MA, USA) for immunoblotting. Homogenates were centrifuged, and tissue lysates were collected and mixed with 6× SDS sample buffer (SSB; 375 mM pH 6.8 Tris, 30% glycerol, 9% SDS, 2.15 M mercaptoethanol, 0.03% bromophenol blue).

### 4.3. RNA Sequencing of Whole Lung RNA and Differential Expression Analysis

RNAseq (n = 6/group) was done by Novogene Corporation Inc. (Sacramento, CA, USA) as previously described [46]. Paired-end reads were aligned to the *Homo sapiens* grch38 reference genome using the Spliced Transcripts Alignment to Reference software version 2.7.10. RNAseq data was deposited in NCBI GEO under accession GSE317056. Differential expression analyses (DEAs) were performed with DESeq2 [47] for the following comparisons: “F-SSc vs. F-NL,” “M-SSc vs. M-NL,” “M-NL vs. F-NL,” and “M-SSc vs. F-SSc.” DESeq2 provided false discovery rate-adjusted *p*-value (q-value) and log2fold change (log2FC) for each gene. Genes were classified as DEGs if they met the thresholds of q-value < 0.05 and log2FC > |0.6|. Genes selected for validation represented a balance of biologically relevant, novel, and data-set driven targets. We included genes with known roles in fibrosis as well as less-studied candidates that showed strong differential expression or network centrality in the current analysis and prior work in our laboratory.

### 4.4. Systems-Level and Impact Pathways Analyses

Systems-level and impact pathways analyses was performed using iPathwayGuide (Advaita Bioinformatics, Ann Arbor, MI, USA) using the same significance criteria described above. This platform identifies pathways that are significantly impacted under different conditions based on high-throughput gene expression data. GO terms with a *p*-value < 0.05 were considered significantly dysregulated. Network analysis was used to identify hub genes, defined as those with the highest number of incoming connections located centrally in the network. In network analysis, centrality is used as a measure of the number of edges connected to a vertex, where the vertices (i.e., genes) with the highest degrees of connectivity (centrality = 1) are assumed to play important roles in the system [18]. A detailed description of their methods can be found in [48]. Causal analysis via the Predicting Upstream Regulators tool in iPathwayGuide was used to identify upstream chemicals, drugs, and toxicants predicted to be absent or insufficient in our F-SSc and M-SSc groups based on the identified DEGs [48]. This analysis tested the hypothesis that changes in gene expression are caused by a deficiency of each chemical, drug, and toxicant in the Comparative Toxicogenomics Database [48,49]. False discovery rate was used to correct *p*-values in multiple comparisons.

To identify the most dominant drugs in the drug–gene network and interpret drug–gene interactions, the miRmapper tool was used [18]. We inputted the lists of upstream drugs and DEGs for female- or male-specific drugs and their respective lists of unique DEGs to create an adjacency matrix of genes to define which gene targets had the greatest degree of centrality. For genes that were downstream of three or more predicted drugs, human-annotated GO queries were performed using ToppFunn from the ToppGene Suite with default settings [50]. GO terms with *p* < 0.05 were visualized in semantic similarity-based scatterplots using Reduce & Visualize Gene Ontology (REViGO), which combines redundant terms into representative terms based on a simple clustering algorithm via semantic similarity measures [51]. The 10 most significant GO terms based on *p*-value were highlighted to indicate overall similarities and spatial organization.

### 4.5. Outgrowth and Cell Culture of the Primary Human Pulmonary Fibroblasts

Primary human lung fibroblasts were cultured using the outgrowth method from lung tissues of donors whose lungs were not used for transplantation and patients with SSc undergoing transplantation, as previously described [31,38,52,53]. Fibroblasts were cultured in DMEM (Corning Inc., Corning, NY, USA) supplemented with 1X antibiotic-antimycotic (AB/AM) (ThermoFisher Scientific, Waltham, MA, USA) and 10% fetal bovine serum (FBS) (Sigma-Aldrich, Saint Louis, MO, USA). Media were changed every other day until fibroblasts reached 90% confluency, after which the fibroblasts were passaged using trypsinization.

To grow cryopreserved fibroblasts, a cryovial was thawed quickly, and its contents were added to 20 mL of complete DMEM in a T75 flask and incubated overnight. The media was changed the next day, and cells were grown to confluency before passaging. Fibroblasts were cultured in 6-well plates in complete DMEM with 10% FBS and 1× AB/AM. Fibroblasts were used in passages 3–8 for qPCR and immunoblotting (n = 6/group). Control (NL) fibroblasts were treated with vehicle (R&D, Minneapolis, MN, USA) or TGFβ1 at 10 ng/nL (R&D, Minneapolis, MN, USA) for 24 h. Cells were washed with PBS and scraped in Trizol (Qiagen, Germantown, MD, USA) for RNA extraction or 2× SSB (125 mM pH 6.8 Tris, 10% glycerol, 3% SDS, 715 mM mercaptoethanol, 0.01% bromophenol blue) for immunoblotting. Total RNA was extracted from fibroblasts using Trizol (Qiagen, Germantown, MD, USA) per the manufacturer’s instructions.

### 4.6. cDNA and qPCR

A NanoDrop Lite spectrophotometer (ThermoFisher Scientific, Waltham, MA, USA) was used to assess RNA quality and quantity. A total of 1 μg of RNA extracted from human lung tissues and fibroblasts was synthesized using the qScript Ultra SuperMix (cat# 95217, Quantabio, Beverly, MA, USA) in a 20 μL cDNA reaction according to the manufacturer’s instructions. cDNA was synthesized on the C1000 Touch Thermal Cycler (Bio-Rad, Hercules, CA, USA) with the following protocol: A. priming stage for 5 min at 25 °C; B. reverse transcription (RT) stage for 20 min at 46 °C; C. RT inactivation stage for 1 min at 95 °C; D. hold at 4 °C.

qPCR reactions were prepared using 5 μL of TaqMan Gene Expression Master Mix (Cat# 4369016, ThermoFisher Scientific, Waltham, MA, USA), 3.5 μL of nuclease-free water, and 0.5 μL of either duplexed *GADPH*/*B2M* housekeeping probes or the target FAM-labeled assay. Amplification was performed on a CFX96 Touch Real-Time PCR System (Bio-Rad, Hercules, CA, USA) using the following cycling conditions: 48 °C for 15 min and 95 °C for 10 min (holding), followed by 40 cycles of 95 °C for 1 min and 60 °C for 1 min. Gene expression was quantified using 2^−ΔCT^ method normalized to *GAPDH* or *B2M*, and statistical analyses were conducted in GraphPad Prism version 10.5.0 (GraphPad Software Inc., La Jolla, CA, USA). The primers utilized are listed in Supplemental File S20.

### 4.7. Immunoblotting

Approximately 100 mg of lung tissue was homogenized in RIPA buffer containing EDTA (Cat# BP-115D, Boston BioProducts, Inc., Milford, MA, USA), supplemented with Halt protease inhibitor (Cat# 78430, ThermoFisher Scientific, Waltham, MA, USA) and sodium orthovanadate as phosphatase inhibitor (Cat# 13721-39-6, ThermoFisher, Scientific, Waltham, MA, USA). The total protein levels were quantified using the Pierce BCA Protein Assay Kit (Cat# 23225, ThermoFisher Scientific, Waltham MA, USA). Whole cell lysates were prepared as described above. Samples were then electrophoresed in SDS-PAGE gels using the ThermoFisher Bolt system following the manufacturer’s instruction. After electrophoresis, proteins were transferred to nitrocellulose membranes (Cat# 10600015, Cytiva, Marlborough, MA, USA) at 300 mA for 2 h and blocked for 1 h at room temperature in 5% non-fat dry milk in TBS-Tween-20.

Membranes were incubated overnight at 4 °C with primary antibodies (Supplemental File S20) followed by secondary antibodies for 1 h at room temperature. Blots were developed using SuperSignal West Pico (Cat# 34578, ThermoFisher Scientific, Waltham, MA, USA) or SignalFire Plus ECL reagent (Cat# 12630S, Cell Signaling, Danvers, MA, USA) and imaged on the iBright750 system (ThermoFisher Scientific, Waltham, MA, USA). Band intensities were quantified using ImageJ version 1.54g and statistical analyses were performed in GraphPad Prism version 10.5.0 (GraphPad Software Inc., La Jolla, CA, USA).

### 4.8. Statistical Analysis

All continuous variables were expressed as the mean ± standard deviation. All statistical analyses were done using GraphPad Prism version 10.5.0 for Windows (GraphPad Software Inc., La Jolla, CA, USA). An unpaired *t*-test was used for comparison between two groups. One-way ANOVA was used for comparison between multiple groups. All *p*-values < 0.05 were considered statistically significant.

## 5. Conclusions

Despite the cross-sectional disease signature of SSc-PF in males having three-fold more DEGs than observed in the analysis of females, both ended with the same SSc-PF transcriptomic signature, with only 32 genes being differentially expressed. This, along with the fact that only 9.19% of DEGs overlapped between the disease-associated transcriptomic changes, suggests that the pathogenesis of SSc-PF varies significantly by sex. Furthermore, our data show there may be continued differentiation of alveolar fibroblasts to inflammatory fibroblasts in males even at end-stage disease. At baseline, there are transcriptomic differences in male normal lungs that predict a “pre-fibrotic” state, suggesting that male lungs are primed to respond to stress stimuli. In addition, there is a sex differential in the TGFβ-induced expression of genes such as *THBS2* and *GNB3* in primary lung fibroblasts. Finally, via causal analysis, we found a significant sex-based difference in potential therapies and the DEGs that these therapies are projected to modulate. Overall, our findings provide insight into the different clinical presentations of men and women with SSc-PF and provide a foundation for future studies exploring sex-specific therapeutic targets for SSc-PF.

## Figures and Tables

**Table 1:** Healthy donor information.

Code	Race	Age	Sex	LG RNA	LG WB	±TGFβ	Cause of Death	Smoker
NL-8	EA	54	M	X			Head trauma	no
NL-9	EA	49	M	X		X	Head trauma	no
NL-14	EA	43	F	X			CVA	yes
NL-16	EA	49	F				Unknown	yes
NL-17	EA	62	F		X		CVA	no
NL-38	EA	37	F		X		N/A; heart/lung transplant due to TPA	no
NL-41	EA	53	F	X			Subarachnoid hemorrhage	yes
NL-43	EA	44	M		X		Unknown; possible bacterial infection	no
NL-45	EA	42	F				Unknown	no
NL-57	EA	60	F	X	X	X	Unknown	no
NL-62	EA	62	F	X			Intracerebral bleeding after CVA	yes
NL-64	EA	49	F		X		CVA	yes
NL-66	EA	63	F			X	Stroke	no
NL-68	EA	55	F		X		CVA	yes
NL-69	EA	38	F		X		Stroke	yes
NL-74	EA	37	M		X		Benign granuloma	no
NL-86	EA	33	M		X		Head trauma	no
NL-87	unknown	50	M		X		Unknown	no
NL-88	EA	52	M		X		CVA	no
NL-96	EA	31	M		X		Anoxia	no
NL-107	EA		F			X		
NL-126	EA	66	M	X		X	Cerebral hemorrhage, stroke	yes
NL-129	EA	51	F	X			Unknown	yes
NL-132	EA	45	M	X			Cardiac arrest	no
NL-133	EA	67	M	X		X	Head trauma	former
NL-140	EA	57	F	X			Stroke	no
NL-144	EA	20	M			X	Head trauma	no
NL-146	EA	58	M	X			CVA	no
NL-152	EA	23	M			X	Drug overdose	no

EA, European American; F, female; M, male, X, used in experiment; CVA, cerebrovascular accident.

**Table 2:** SSc-PF donor information.

Code	Race	Age	Sex	LG RNA	LG WB	Diagnosis; Pathology	Smoker	Subtype	FVC (%)	mPA (mm Hg)
SSc-8	EA	26	F		X	SSc-PF	no	Sine	28	13
SSc-11	EA	45	M	X	X	SSc-PF	no	Diffuse	30	23
SSc-12	EA	47	M		X	SSc-PF	no	Sine	33	24
SSc-19	EA	37	F	X		SSc-PF with mild HTN; UIP/NSIP	no	Diffuse	18	26
SSc-23	EA	51	F		X	SSc-PF	yes	Limited	36	22
SSc-24	EA	45	M	X	X	SSc-PF	no	Unknown	26	23
SSc-25	EA	52	F	X		SSc-PF; UIP/granuloma	yes	Diffuse	23	20
SSc-26	EA	57	M	X	X	SSc-PF	no	Diffuse	33	24
SSc-27	EA	42	F	X	X	SSc-PF; UIP	yes	Diffuse	42	15
SSc-29	EA	60	F	X		SSc-PF	no	Limited	61	23
SSc-30	EA	59	F	X		SSc-PF; UIP	no	Unknown	39	19
SSc-38	EA	49	F	X		UIP, hyaline membrane	yes	Limited	26	21
SSc-43	EA	62	M	X		SSc-PF with HTN	No	Unknown	54	27
SSc-53	EA	46	M	X		SSc-PF; UIP with HTN; zones of NSIP	yes	Unknown	52	22
SSc-66	EA	51	M	X		SSc-PF	no	Limited	70	25
SSc-67	EA	68	F	X		SSc-PF	no	Unknown	52	21
SSc-81	EA	67	M		X	SSc-PF with unknown HTN status	no	Unknown	25	
SSc-82	EA	54	F		X	SSc-PF	no	Unknown	41	27
SSc-83	EA	52	F		X	SSc-PF	yes	Unknown	26	25
SSc-87	EA	64	M	X	X	SSc-PF	yes	Unknown	56	23
SSc-113	EA	67	M		X	SSc-PF	former	Limited	60	24

WB, Western blot; EA, European American; F, female; M, male; X, used in experiment; UIP, usual interstitial pneumonia; NSIP, nonspecific interstitial pneumonia; HTN, hypertension.

## Data Availability

The data presented in this study are openly available in GEO at https://www.ncbi.nlm.nih.gov/geo/, reference number GSE317056.

## Supplementary Materials

The following supporting information can be downloaded at: https://www.mdpi.com/article/10.3390/ijms27104363/s1.

## Abbreviations

The following abbreviations are used in this manuscript:

SSc	Systemic sclerosis
SSc-PF	Systemic sclerosis-associated pulmonary fibrosis
FDA	Food & Drug Administration
ECM	Extracellular matrix
TGFβ	Transforming growth factor β
PDGF	Platelet-derived growth factor
DEG	Differentially expressed gene
NL	Normal lung
F	Female
M	Male
DEA	Differential expression analysis
GNB3	G protein subunit β3
GO	Gene ontology
qPCR	Quantitative polymerase chain reaction
KEGG	Kyoto Encyclopedia of Genes and Genomes
THBS	Thrombospondin
GABA	Gamma-aminobutyric acid
yo	Years old
FBS	Fetal bovine serum
