# Supplementary figures and images for: Sex Disparity in Systemic Sclerosis-Associated Pulmonary Fibrosis
Source: Int J Mol Sci. 2026 May 14;27(10):4363. doi: 10.3390/ijms27104363 (PMC13208012; doi:10.3390/ijms27104363)

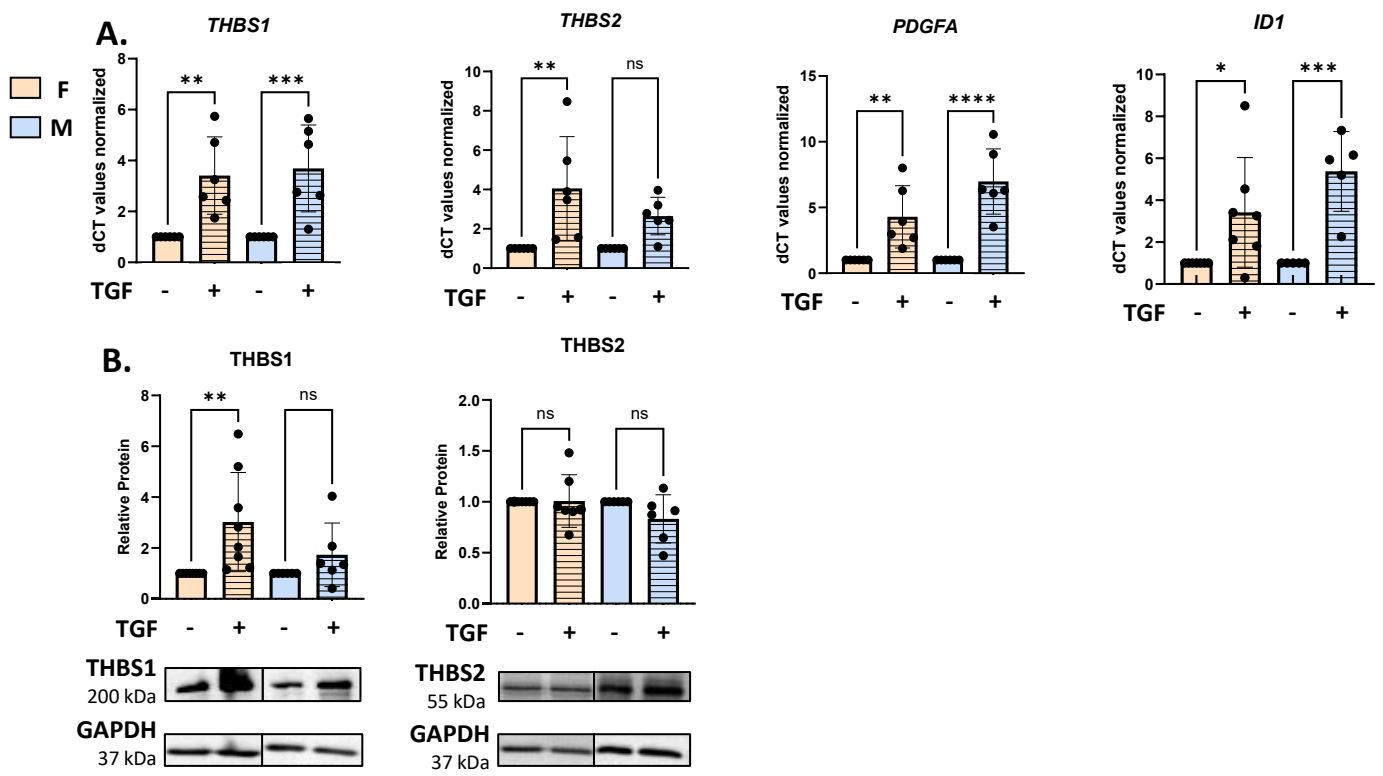

Supplement: Supplementary file 1 [file ijms-27-04363-s001.zip › Galimba et al Supplemental Files/Supplemental File 13_Effect of TGF on fibroblast DEGs unique to females.pdf]

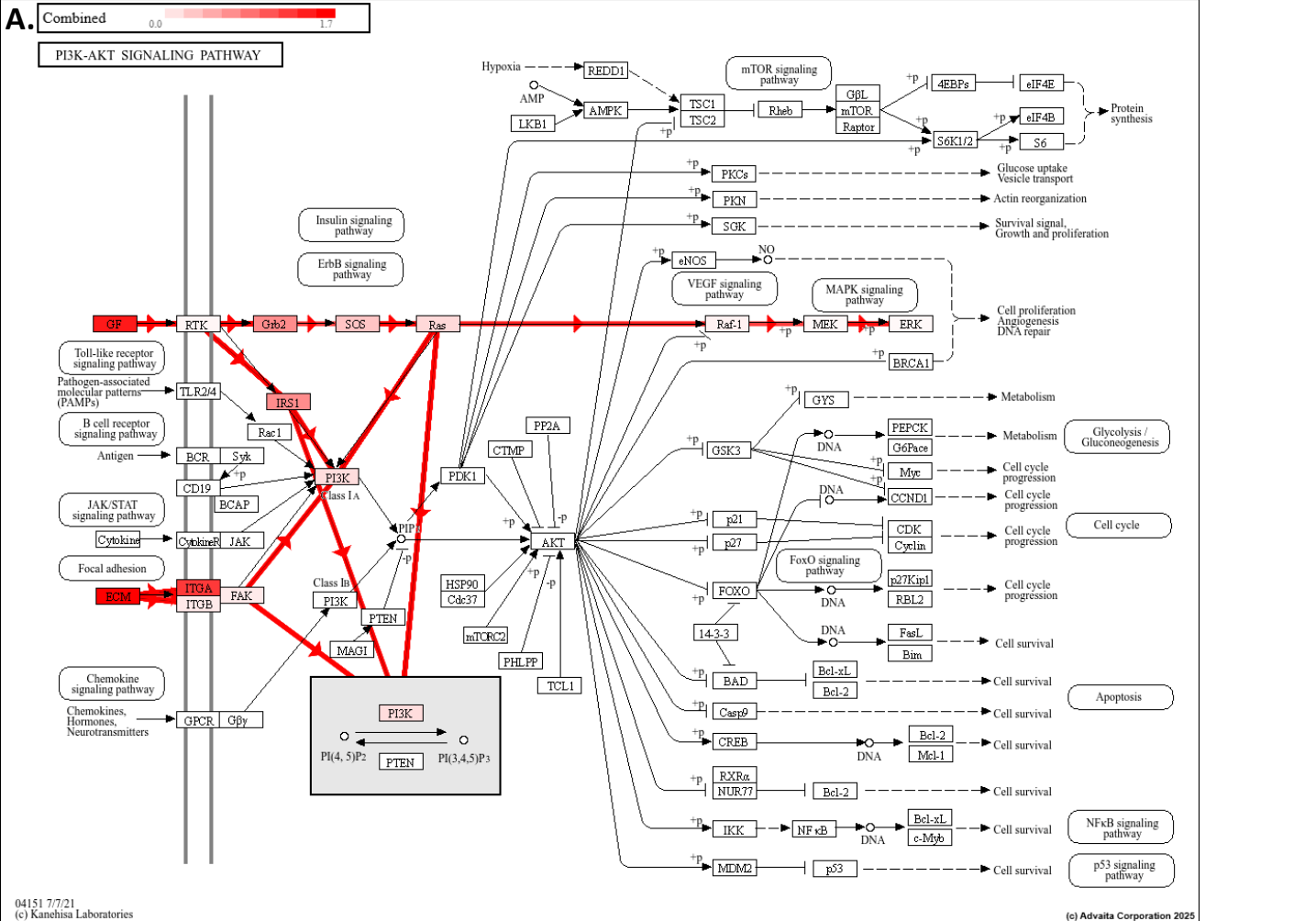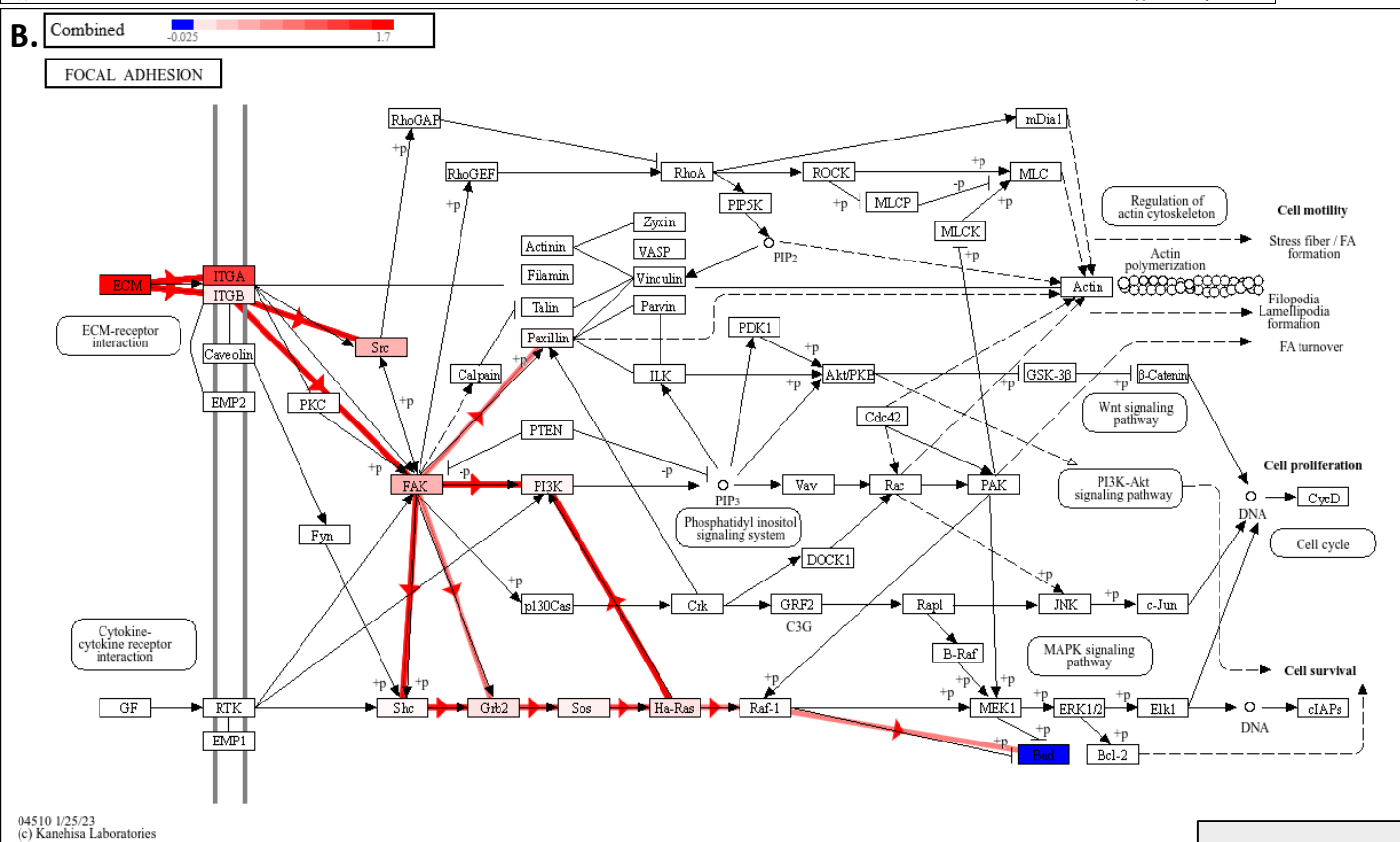

Supplement: Supplementary file 1 [file ijms-27-04363-s001.zip › Galimba et al Supplemental Files/Supplemental File 10_Enrichment of select pathways in males NL.pdf]

**A.**

**insoluble COL6A6**

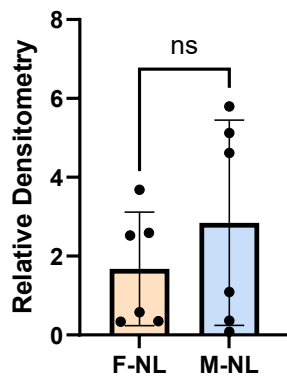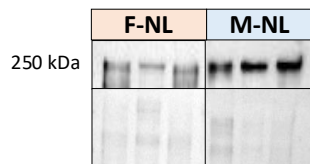

**B.**

**ANGPT1**

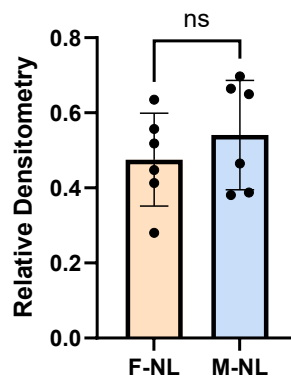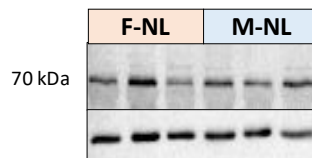

**C.**

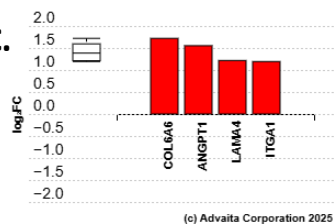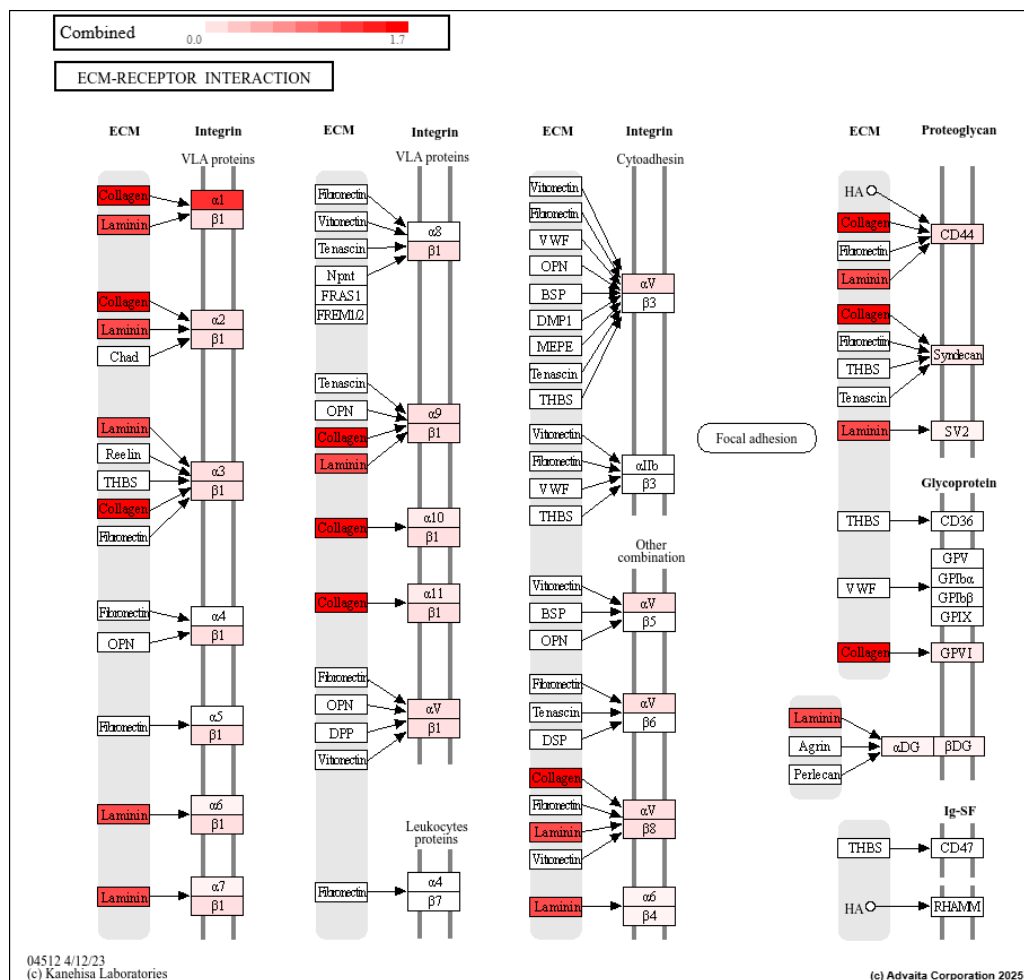

Supplement: Supplementary file 1 [file ijms-27-04363-s001.zip › Galimba et al Supplemental Files/Supplemental File 09_Enrichment of ECM interaction in males NL.pdf]
